# Richmond Academy of Medicine and Surgery

**Published:** 1896-11

**Authors:** 


### Society Reports.

### RICHMOND ACADEMY OF MEDICINE AND SURGERY.

[CONTINUED FROM OCTOBER ISSUE.]

*Query.*—What is the matter? The only pronounced symptom is the pain. She will not voluntarily change her position, and as a result, she has a bed-sore.

What is the cause of the rise of temperature? Under the use of turpentine, the condition has improved somewhat. There may be an abscess of the kidney. He had suspected the pain to be hysterical. She had been taking a great deal of morphine—in one day, a grain and a half hypodermically. Since being at the hospital, the doctor has tried to do away with its use. The patient has taken several remedies for the relief of pain, usually falling back on morphine. As an experiment, a syringeful of pure water was injected, but in a half hour, complaint of great pain obliged administration of a one-fourth grain of morphine. The doctor thought a great deal of the pain was due to the fear of it on being moved.

There were no sweats, no hectic; she is not sensitive to light pressure. Examination by her attending physician elicited great complaint of pain; but in ten minutes, he could manipulate and not get much response.

Dr. Hodges wished to suggest that the first case was thrombus with suppuration, complicated or not by typhoid. He thought the second case more than simple hysteria. If there were no disease of the pelvic organs, it might be neuralgia or neuritis with after-developed hysteria or fever.

Dr. Upshur replied that there was no neuritis.

### IS THERE A TYPHO-MALARIAL FEVER?

The Secretary reported a case, not because of uniqueness, but as contributing to the settlement of the much mooted existence of typho-malarial fever.

T. H. P., male, age 36, more than three weeks ago was taken sick with what looked like cholera morbus. In four days a chill came on, followed by sweating and fever; thereafter, for four or five days, there were two and three chills at irregular intervals daily; the temperature one morning reaching 106°, falling to 102° upon the application of a cold bath. Thirty grains of hydrochlorate of quinine were administered in twelve hours, and as a result chills ceased, but there remained a persistent fever, which was stationary in the morning, rising in the afternoon, and accompanied by nearly all the symptoms which justified a diagnosis of typhoid fever. He entered his fourth week of sickness to-day, but his temperature has been normal for four or five days in the morning, with exacerbation in the afternoon. Chills came on again last Friday, and with the exception of Sunday, he has had them daily at irregular intervals, and sometimes two in twenty-four hours.

Dr. R. F. Williams made a blood examination and found absolutely no plasmodium, but a marked leucocytosis, which argues a favorable result.

Drs. Gordon and Deaton saw the case in consultation, and agreed that the trouble is *pure typhoid*.

Dr. Wm. S. Gordon said, regarding this case, that it was undoubtedly typhoid. If it had been typho-malaria, when the chills were stopped the fever should have ceased, and the blood examination further verified the diagnosis. He reported the case of a lady confined two weeks ago. He had ceased his attention, when on the twelfth day she was seized by a violent chill at 4:30 p. m., and again at 11 p. m. Friday last, there was another at 11 a. m. Quartan fever being suspected, he gave quinine, but there ensued a chill at 11 p. m., followed by high fever and sweating. There was no trouble with the breast that morning; the lochia were free from odor, and everything relating to the uterus was normal. At night, the breast was affected and lead-water was advised. The next morning mammitis had come on—no suppuration. The breast trouble did not ensue until the fourth day. It was relieved. Yesterday, the patient was entirely free from fever. She was thoroughly cinchonized. Remembering the influence on the mind of the time of attacks, he endeavored to take hers from under its influence by mental diversion, anecdotes, etc.; but at 4:30 p. m. the chill came. If she had not been under the influence of quinine, she would in all probability have had another last night. The case, a double quotidian of the quartan type, is one the doctor has never seen before.

As to the cause. The house is surrounded by flowers, grown over with vines; has, necessarily, decaying vegetation in the garden; flowers in the rooms. These, with moisture and heat, furnish all the etiology desired.

### FACIAL PARALYSIS FOLLOWING MUMPS, ENLARGED ONE HALF OF THYROID, ETC.

Dr. JACOB MICHAUX described a case of facial paralysis. Girl, age 17; personal history good. A short time ago he was told she had mumps. The left side of the face and lips were decidedly swollen, having the stiff appearance indicating loss of motion, which, indeed, was the case. There was tenderness under the ear, but he was struck by the absence of swelling in front of the ear (which usually occurs in mumps) and by the appearance of the lids, mouth and side of the face. The tissues on the affected side were tender, especially at the angle of the jaw.

On complaining of her throat, he examined it and found enlargement of the right lobe of the thyroid decided, but none of the left.

She was treated at home until acute symptoms subsided, with iodide of potassium and corrosive chloride of mercury. After she was able to be up and about, faradic electricity was applied to the affected side, and both it and galvanic, especially the latter, to the enlarged gland.

The doctor wished to know if there was any connection between the two conditions, or the parts. If so, what?

The girl was not positive as to the existence of thyroid enlargement before the facial symptoms. There was no sore throat, but some temperature.

### EXTRA-UTERINE CAVITY.

Dr. MICHAUX's second case was an unusual condition of the uterine canal, which he has seen twice. Both were in cases of abortion—one at two, the other at four months. In the latter, on introduction of the finger into the os, he found a cavity, which, as well as he could make out, was three inches in diameter. At first, he thought he had a case of placenta prævia, but on passing the finger through, he discovered the fœtus and placenta, which were duly delivered. In the other case the canal was smaller.

